# Pharmacological Insights and Clinical Applications of Ciprofol: A Narrative Review

**DOI:** 10.7759/cureus.68034

**Published:** 2024-08-28

**Authors:** Shubham Petkar, Amol Bele, Vishnu Priya, Dushyant Bawiskar

**Affiliations:** 1 Anaesthesiology, Jawaharlal Nehru Medical College, Datta Meghe Institute of Higher Education and Research, Wardha, IND; 2 Sports Medicine, Abhinav Bindra Targeting Performance, Bangalore, IND

**Keywords:** propofol, anaesthesia, sedative, gamma-aminobutyric acid, ciprofol

## Abstract

Ciprofol (HSK3486) is a novel intravenous anaesthetic developed as an alternative to propofol, offering a safer and more effective option in anaesthesia. It works primarily by modulating the gamma-aminobutyric acid (GABA) receptors in the central nervous system, leading to sedation and hypnosis. Ciprofol’s unique pharmacological properties include a rapid onset of action, shorter duration, and reduced cardiovascular and respiratory depression compared to propofol, making it particularly suitable for outpatient and day surgery procedures. Molecular changes in ciprofol appear to be superior to those of other cibenzolines; it is more potent and has a stable hemodynamic effect. It has been used in primary surgery, inpatients and outpatients, and even for sedation of intensive care patients. The reported clinical data indicate that ciprofol is a powerful sedative that is characterised by a high-enough speed of emergence from the state of anaesthesia, which is necessary for outpatient conditions and intensive operating modes. It can be considered a new and important perspective in the technology of intravenous anaesthetics with its improved pharmacological characteristics and clinical effects. With the further accumulation of clinical data, ciprofol will undeniably become an essential agent in today’s anaesthetic practice and contribute to an enhancement of healthcare efficiency by providing a more secure approach to numerous kinds of surgical interventions. The purpose of the current study is to provide a review of the pharmacology and clinical use of ciprofol, a new intravenous anaesthetic agent. Various studies demonstrate the functionality and safety profile of ciprofol, which solidifies it as a potential contender for propofol. Regarding respiratory depression, hypoxemia, and injection pain during hysteroscopy, ciprofol was shown to be a relatively safer option than propofol. Ciprofol can, therefore, be recommended for intravenous anaesthesia because of its effectiveness and safety, which has been clearly demonstrated. Randomised trials uniformly report the ability to achieve quicker onset of sedation and lower risk with the agent compared to propofol. These findings imply that ciprofol has many benefits concerning a variety of applications in patients due to a lower rate of adverse reactions and increased patient comfort.

## Introduction and background

Ciprofol (HSK3486) was developed in the early 2010s as a novel intravenous anaesthetic aimed at improving the safety profile of propofol. Initial research began in the early 2000s, leading to successful preclinical studies that demonstrated its efficacy and safety. Clinical trials conducted between 2017 and 2020 confirmed its potential, resulting in regulatory approval and its introduction into anaesthesia practice, particularly in China, where it has been widely adopted. However, ciprofol has a somewhat different chemical structure derived from propofol, but it increases its quality by enhancing its pharmacokinetic and pharmacodynamic properties. This design intends to address the drawbacks linked to propofol, particularly the side effects like hypotension and the pain experienced during injection, making this design safer and more tolerable when it comes to patients going through several medical procedures [1,2].

Pharmacologically, ciprofol acts mainly through enhancing the effect of gamma-aminobutyric acid (GABA) receptors in the central nervous system. It promotes the immediate onset and the sustainment of the sedation or anaesthesia, similar to the case with propofol. However, molecular changes in ciprofol appear to be superior to those of other Cibenzolines; it is more potent and has a stable hemodynamic effect. However, studies conducted in animals have shown that ciprofol causes less injection pain and cardio-depressing effect than its counterpart; hence, it should attract clinicians' attention in practice [3,4].

Ciprofol has been used in primary surgery, inpatients and outpatients, and even for sedation of intensive care patients. The reported clinical data indicate that ciprofol is a powerful sedative that is characterised by a high-enough speed of emergence from the state of anaesthesia, which is necessary for outpatient conditions and intensive operating modes. Also, its pharmacokinetic properties reflect a short distribution half-life and a very short clearance half-life. This factor makes it quite predictable dosing and easy to titer, especially during procedures [5]. All these attributes not only improve the condition of the patient and their safety but also optimise the process of the healthcare providers' work [6,7].

Objective of the study

The purpose of the current study is to provide a review of the pharmacology and clinical use of ciprofol. This review will explain the differences in the pharmacokinetic and pharmacodynamic profiles of ciprofol. In addition, this article aims to review clinical information in order to evaluate the outcomes of ciprofol exposure in view of safety/concerns, efficacy/potential, and medical uses/treatment, in an attempt to outline the directions of further research and the clinical application of ciprofol in modern anesthesiology.

## Review

Pharmacokinetics

Absorption and Distribution

Ciprofol, similar to propofol, is administered intravenously, ensuring complete bioavailability. After administration, ciprofol rapidly distributes into the central nervous system (CNS) due to its high lipophilicity. This rapid CNS penetration is responsible for its swift onset of anaesthetic effects. The volume of distribution (Vd) of ciprofol is substantial, indicating extensive distribution into body tissues, particularly adipose tissue. This characteristic is typical of lipophilic drugs, allowing for quick CNS action but also contributing to a prolonged elimination phase due to redistribution into peripheral compartments [8].

Metabolism

Ciprofol is primarily metabolized in the liver, with the major metabolic pathway being hydroxylation followed by conjugation with glucuronic acid, resulting in the formation of inactive metabolites. Studies suggest that the metabolism of ciprofol may involve cytochrome P450 enzymes, similar to propofol, although specific enzymes have not been as clearly defined in the literature. The rapid metabolism of ciprofol contributes to its short duration of action and favourable recovery profile, as it is quickly cleared from the bloodstream after cessation of administration [9,10].

Elimination

The elimination half-life of ciprofol is relatively short, ranging from 2 to 4 hours, depending on patient-specific factors such as hepatic function and body composition. Ciprofol is predominantly excreted in the urine as an inactive metabolite, with minimal unchanged drugs being excreted. The drug's high clearance rate, which is similar to that of propofol, prevents accumulation in the body, even with prolonged infusions. This characteristic is crucial for maintaining a stable level of anaesthesia during surgical procedures and for ensuring rapid recovery post-operatively [11,12].

Pharmacodynamics

Mechanism of Action

Ciprofol exerts its anaesthetic effects primarily through positive modulation of the GABA type A receptors in the CNS. By enhancing GABA_A receptor activity, ciprofol increases chloride ion influx into neurons, leading to hyperpolarization and subsequent inhibition of neuronal activity. This mechanism is similar to that of propofol but with slight modifications due to ciprofol's chemical structure, potentially resulting in different binding affinities and potencies [13].

Onset and Duration of Action

Ciprofol is characterized by a rapid onset of action, typically within 30 to 60 seconds following intravenous administration. This rapid onset is beneficial in clinical settings requiring quick induction of anaesthesia. The duration of ciprofol's anaesthetic effect is short, generally lasting about 5 to 10 minutes after a single bolus injection. This brief duration of action is largely due to the drug's rapid redistribution from the CNS to peripheral tissues and its swift hepatic metabolism. Such pharmacodynamics make ciprofol suitable for short procedures or as an induction agent before transitioning to other maintenance anaesthetics [14].

Clinical Efficacy and Safety Profile

Ciprofol has been shown to be more potent than propofol, meaning that lower doses are required to achieve comparable levels of sedation or anaesthesia. This increased potency may reduce the overall drug load and potentially lower the risk of dose-related adverse effects. However, as with all anaesthetics, ciprofol can cause dose-dependent respiratory and cardiovascular depression. The literature indicates that while ciprofol may have a slightly better safety profile than propofol, with less-pronounced hypotension and respiratory depression, careful monitoring remains essential, especially in patients with underlying cardiovascular or respiratory conditions [15].

Clinical efficiency in various surgical settings

Jenshan Lin conducted a study on 60 women undergoing daytime hysteroscopy, who were divided into two groups and given an initial dose of ciprofol at 0.4 mg/kg or an initial dose of propofol at 2 mg/kg. This was given 2 minutes after an intravenous injection of sufentanil 0.15 ug/kg. The ED50 (effective dose for 50% of the population) values for ciprofol and propofol in inhibiting the reaction during hysteroscopy dilatation were determined. Ciprofol had a significantly lower incidence of side effects, such as respiratory depression, hypoxemia, and injection pain, than propofol during the hysteroscopy sedation procedure. This implies that ciprofol may provide a safer sedation option for outpatient hysteroscopic procedures [16]. Kuo-Chuan Hung et al. conducted a thorough search of multiple databases to compare the efficacy and safety of ciprofol and propofol for anaesthetic induction and non-intensive care unit (ICU) sedation. The study concluded that ciprofol and propofol were equally effective for sedation and anaesthetic induction, with ciprofol having lower risks of hypotension and pain on injection. The findings indicate that ciprofol may be an acceptable substitute for propofol in clinical practice [17].

Hudaib Tajamal et al. conducted a randomised controlled trial to compare ciprofol and propofol for induction and maintenance of general anaesthesia in patients undergoing surgery. Ciprofol demonstrated advantages in pain management during the induction phase, potentially improving patient comfort during surgery. Ciprofol and propofol have similar efficacy and safety profiles for anaesthesia induction and maintenance in adult patients undergoing surgery [4]. The clinical trial data were synthesised to assess the efficacy and safety of ciprofol in various scenarios. Ciprofol has a slightly higher therapeutic index in rats than propofol, indicating a larger safety margin. Furthermore, clinical trials have shown that ciprofol has advantages such as improved tolerance, higher sedation satisfaction scores, and a lower incidence of adverse reactions, particularly pain reduction following injection [3].

Jing Xu et al. did a study. The trial included 135 women aged 18 to 65 years who were scheduled for ambulatory gynaecological procedures. Its purpose was to assess the efficacy and safety of ciprofol for sedation in outpatient gynaecological procedures. Ciprofol was found to be non-inferior to propofol for sedation in outpatient gynaecological procedures, with both drugs achieving a 100% completion rate without the need for additional anaesthetics. Ciprofol had a better safety profile than propofol, with fewer treatment-emergent adverse events and injection pain in patients who received ciprofol sedation [18]. Gongchen Duan et al. used a randomised, controlled trial design to evaluate the safety and effectiveness of various doses of ciprofol in inducing general anaesthesia in elderly patients. The study involved 105 elderly patients undergoing elective surgery. The study compared three different ciprofol doses (0.2 mg/kg, 0.3 mg/kg, and 0.4 mg/kg) and discovered that the 0.3 mg/kg dose provided good safety and efficacy in anaesthesia induction in elderly patients. Adverse events such as hypotension, hypertension, bradycardia, tachycardia, hypoxemia, and injection discomfort were recorded across all dosage groups. The probability of complications was significantly greater in the lower (0.2 mg/kg) and higher (0.4 mg/kg) dose groups than in the 0.3 mg/kg group [19].

Y Man et al. used a randomised, double-blind controlled trial design to evaluate the efficacy and safety of ciprofol in anaesthesia for gynaecological day surgery. The study included 128 patients who underwent gynaecological day surgery under general anaesthesia. The study included 128 patients who underwent gynaecological day surgery under general anaesthesia. The study findings support that ciprofol is as effective as propofol in anaesthesia for gynaecological day surgery while also demonstrating a lower incidence of adverse events [20].

The cost-effectiveness of using ciprofol, a sedative, has been studied in various contexts. Studies have shown that ciprofol can be a cost-efficient alternative in medical treatments, such as bronchoscopy in tuberculosis patients, and in the treatment of serious infections, where it can lead to considerable cost savings compared to traditional intravenous therapies. For instance, in the case of bronchoscopy in tuberculosis patients, the median effective dose of ciprofol varied based on gender and age groups, highlighting the importance of personalised dosing for optimal outcomes. Additionally, the use of oral ciprofol has been found to be effective and cost-efficient, achieving high clinical cure rates and significant cost avoidance compared to intravenous antimicrobial therapy. These findings underscore the potential economic benefits of incorporating ciprofol into medical protocols for various conditions [21,22].

Comparison with other drugs

Ciprofol is one of the recently introduced non-barbiturate soothing agents, and its anaesthetic profile has been compared with other drugs in various trials. In a study concerning neurosurgical persons, ciprofol was found to be non-inferior to propofol in terms of effectiveness in intraoperative neurophysiological monitoring during microvascular decompression surgical procedures with added advantages that include the alleviation of injection pain and the enhancement of haemodynamic stability [23]. One study compared ciprofol with propofol in patients who were undergoing painless endoscopic procedures, including gastroscopy, and the results showed that both drugs were effective in the prevention of PONV. However, propofol had a better antiemetic effect in comparison with ciprofol [24]. Nandha Kumar Durai Samy et al. used a comprehensive review approach to find alternative intravenous anaesthesia options to ciprofol. The review paper on intravenous anaesthesia options concludes that while ciprofol has advantages such as rapid onset and predictable offset, concerns about its safety profile and individual variability in response have led to the search for alternative agents. The paper concludes that while ciprofol has advantages such as rapid onset and predictable offset, concerns about its safety profile and individual response variability have led to the search for alternative intravenous anaesthesia options. Established agents such as propofol, etomidate, ketamine, and dexmedetomidine were discussed as viable alternatives to ciprofol, each offering unique properties and potential benefits [16,25]. Furthermore, in another study concerning gynaecological ambulatory surgery, ciprofol showed an anaesthetic impact equivalent to that of propofol but with a relatively lower rate of adverse reactions [26].

Disadvantages

Unlike many marketed drugs in developing countries, ciprofol has proved useful in different areas of medicine and surgical procedures such as cardiac surgery, painless gastrointestinal endoscopy, and as an induction agent in elderly patients undergoing minor surgery. However, it has been established that ciprofol is efficient and safe to use; however, it has some disadvantages. The previously mentioned papers also indicate that when administered at higher doses, ciprofol raises the risks of side effects like hypotensive effects, respiratory depression, and injection pain [27,28]. Besides, receiver operating characteristic analysis also reinforces ciprofol’s rapid onset and elimination, but more investigation to prove the sedation effect and safety of ciprofol in ICU patients under mechanical ventilation is required [29-31]. It is crucial to consider several factors when it comes to its dosage and the patients undergoing the procedure to eliminate potentially negative outcomes. No studies have been done to compare the costs and benefits of ciprofol and other anaesthetics. Although ciprofol is currently about three times more expensive than propofol, we think that as the medication is promoted, its cost will drop. While ciprofol is a good substitute for adults, it may also be beneficial for children or patients with other particular conditions. To definitively say whether ciprofol, like propofol, has a wide range of applications for treating seizures, acting as a neuroprotector, and acting as an antiemetic, more research is required. Currently, ciprofol has received attention from researchers in other countries [32-35].

Discussion

The evident impact in numerous clinical trials, specifically in German and Japanese investigations, demonstrates the functionality and safety profile of ciprofol, which solidifies it as a potential contender for propofol. Regarding respiratory depression, hypoxemia, and injection pain during hysteroscopy, ciprofol was shown to be a relatively safer option than propofol in the study done by Lin et al. [16]. This is in line with the observations made by Hung and others, who worked on the meta-analysis of randomised clinical trials and the results that indicated that ciprofol and propofol were comparable in terms of sedation and anaesthetic induction. In contrast, ciprofol revealed lower hazard ratios of hypotension and injection pain, pointing to its better safety profile. Taken together, these studies support the possibility of using ciprofol to improve the patient’s comfort and safety during the process of anaesthesia [17].

In support of these studies, Tajamal et al., in their similar randomised controlled trial with ciprofol and propofol conducted on patients undergoing surgery, report the comparable efficacy and safety of ciprofol in the use of anaesthesia induction and maintenance. Most importantly and often a clinically relevant finding, ciprofol appears to result in better pain control during induction, which could enhance the patient's comfort and the ensuing procedure's outcome [4]. Organising the clinical trial synthesis by Lu et al., the authors pointed out that ciprofol demonstrated a better therapeutic index and tolerance with fewer side effects and higher sedation satisfaction scores compared to propofol. Such findings imply that ciprofol may provide a better therapeutic ratio and a better perception of the patients [3].

Thus, ciprofol has a relatively safe profile; however, further research is still required, especially in certain subgroups of patients. In the prospective randomised study of Xu et al. about outpatient gynaecological procedures, ciprofol proved to be non-inferior to propofol with significantly fewer treatment adverse events and lesser injection pain, which strengthens its appropriateness in day cases. Conversely, Duan et al.'s study on elderly patients highlighted that the dose requirement for ciprofol is an important factor in maintaining efficacy and safety since different doses of ciprofol can have varying impact. This explains why the proper dosing regimens must be set for each patient [18,19].

## Conclusions

Ciprofol can, therefore, be recommended for intravenous anaesthesia because of its effectiveness and safety, which has been clearly demonstrated. Randomised trials uniformly report the ability to achieve quicker onset of sedation and lower risk with the agent compared to propofol. These findings imply that ciprofol has many benefits concerning a variety of applications in patients due to a lower rate of adverse reactions and increased patient comfort. Nevertheless, the careful continuation of research in ciprofol application and monitoring the selection and dosage of the drug for patients will be essential for attaining the true potential of the drug and its proper utilisation across various patient populations.
